# A basis for prenatal diagnosis of Haemophilia-A in Pakistani patients

**DOI:** 10.12669/pjms.38.8.6524

**Published:** 2022

**Authors:** Muhammad Arif Sadiq, Suhaib Ahmed, Muhammad Afzal, Sunila Tasfeen

**Affiliations:** 1 Dr. Muhammad Arif Sadiq, FCPS. Department of Pathology, Riphah International University, Islamabad, Pakistan; 2 Dr. Suhaib Ahmed, FCPS, PhD. Department of Pathology, Riphah International University, Islamabad, Pakistan; 3 Dr. Muhammad Afzal, PhD. Faculty of Basic Medical Sciences, Riphah International University, Islamabad, Pakistan; 4 Dr. Sunila Tashfeen, FCPS. Department of Pathology, Army Medical College, Rawalpindi, Pakistan

**Keywords:** Haemophilia-A, Intron 13, Recurrent F8 gene variant

## Abstract

**Objective:**

To screen Haemophilia-A patients for five known recurrent F8 gene variants and to analyze CA repeats in intron 13 of F8 gene in the mother and the affected child pairs.

**Methods:**

In this descriptive cross sectional study, 80 unrelated pairs of “mother and son” affected by Haemophilia-A were recruited as subjects. After collection of samples DNA was extracted using the Chelex (Bio-Rad, USA) method. Five known F8 gene variants were screened in the mothers and the affected sons by the allele specific PCR. The amplified products were separated on 2% agarose gels.CA repeats in intron 13 of the F8 gene in the mother and the affected child pairs were analyzed by Sanger sequencing method. The CA repeat alleles were used to look for the feasibility of linkage based diagnosis of Haemophilia-A in the affected families. The data were analyzed using Statistical Package for Social Sciences (SPSS) version 22.0.

**Results:**

In the 80 subject “mother and son” pairs a recurrent F8 gene variant was found in 32 pairs (40%). The most recurrent variant c.6869G>A was seen in 12 (15%). Linkage based analysis of the CA repeats in intron 13 was found to be informative in 29 (36.2%) mother-son pairs.

**Conclusion:**

The five known Haemophilia-A disease causing variants were found in 40% of the Pakistani Haemophilia-A patients. The five recurrent F8 gene variants and the CA repeats in intron 13 of F8 gene can provide a comprehensive basis for carrying out prenatal diagnosis and carrier screening in majority of the Pakistani Haemophilia-A families.

## INTRODUCTION

Von Willebrand disease (VWD) is the most common bleeding disorder due to coagulation abnormality. Haemophilia is also among the commonest coagulation factor deficiency bleeding disorders.^1^ The incidence of Haemophilia-A is 1 in 5000 live births approximately.^2^ Its transmission is X linked recessive and carries no variation in relation to ethnicity and geographic distribution. Haemophilia-A gene carrier female transmits the disease to their male offspring.^3^ This disease can lead to several complications such as arthropathy and life threatening bleeds.^4^ The disease is not curable and the patients of hemophilia A require lifelong treatment.

The gene encoding factor VIII is located on long arm of X chromosome and is approximately 190 kb in size. Most common variants leading to Haemophilia-A includes inversions, point variant, small or large deletions and insertions.^5^ The detection of disease or carrier status is either through direct variant analysis or linkage based analysis. Direct variant analysis is the most effective method of diagnosis since it carries almost 100% accuracy and more than 95% informativeness in families.^6^ Linkage based analysis is used by many laboratories in developing countries. The informativeness of short tandem repeat (STR) in linkage analysis of Haemophilia-A is around 80%.^7^ Intron 13 CA repeats polymorphism is one of the most informative intragenic markers in Haemophilia-A. Other intragenic markers for linkage analysis include intron 22 STR, Hind III, and Bcl I.^8^

This aim of this study was to provide a comprehensive basis for carrying out prenatal diagnosis in Pakistani Haemophilia-A patients. We screened the five previously reported Haemophilia-A disease causing variants in the Pakistani patients. We also analyzed CA repeats in intron 13 of factor VIII (F8) gene. Keeping in view the financial constraints and low affordability of Haemophilia-A families especially in developing countries like Pakistan, this study will help in providing a cost effective prenatal diagnosis in a significant number of Haemophilia-A families.

## METHODS

This study was conducted at research laboratory of Riphah International University Al-Meezan Campus Rawalpindi from January 2020 to January 2022. A total of 80 unrelated “mother and son” pairs affected by Haemophilia-A were studied. The subjects (son) selected were registered in Hemophilia Patients Welfare Society Rawalpindi chapter for factor VIII replacement therapy. Ethical approval was granted by institutional ethical review committee of Riphah International University, Islamabad, Pakistan (Ref # Riphah/IRC/19/0362). Sampling technique employed was non probability purposive sampling. Written informed consent was obtained from all participants of the study. A 5ml peripheral blood sample in EDTA anticoagulant was collected each from the mother and affected son. Families having affected son with Haemophilia-A confirmed by excluding inhibitors and factor VIII levels were enrolled in the study. Patients with suspected Haemophilia-A or with any other bleeding disorder were excluded from the study.

Genomic DNA was extracted from blood samples using Chelex (Bio-Rad USA) method of DNA extraction.^9^ DNA amplification was performed in a total volume of 25 μl of PCR reaction mixture. The reaction mixture contained the DNA template (100 ng/μl) and12.5 μl of Dream Taq Green PCR Master Mix (Cat. # 1081, Thermo Scientific). In addition, one μl primer mix (10 pmol/μl) and PCR grade water were added to make the required volume.

Five recurrent F8 gene variants^10^ previously reported in Pakistani population were selected to screen by the allele specific PCR. The primers used for recurrent F8 gene variants and intron 13 CA repeat analyses in F8 gene were as per Table-I.

**Table-I:** Primers for recurrent F8 gene variants and Intron13 CA repeats.

F8-Variants	Primer ID	Sequence 5’to 3’	nt
c.1786T>C	F8T>C-F	GATTAGATATATCACATGCATGCCATCGC	29
	F8T>C-RM	AGCTTCGGTTCTCATCAAATACCGG	25
c.1694G>A	F8G>A-FM	TTAATATGGAGAGAGATCTAGCTTCCGA	28
	F8G>A-R	AGGCACGTTTACTACGTGAAACTCAAA	27
c.195C>G	F8C>G-F	CAACTTCAAATTTGCCTCCTTGCTA	25
	F8C>G-RM	CCGTGAATTCTACAAACAGAGTCTTTATC	29
c.6869G>A	F8G>A-FM	CCAGCAGTCAAGATGGCCATCATTA	25
	F8G>A-R	CAGAATGCCTTTTACAATGTCTTGCTACCA	30
c.6972C>T	F8C>T-F	AGCAGGGTGCTCTTAATTCTAGATGTCCC	29
	F8C>T-RM	AACTCTGGGGGTGAATTCGACGA	23
***Sequencing Primers for Intron 13***			
Intron 13	Int.13-F	GCCTTAAACGTCTGCATTGTCTA	23
	Int.13-R	AGACAAAGCCTTCCTAGATGTGAC	24

Thermal cycling conditions for five recurrent F8 gene variants using PCR machine (Major cycler, USA) were as follows: an initial denaturation at 94ºC for five minutes was followed by 30 cycles of denaturation at 95ºC for 30 seconds, annealingat 60 ºC for 30 seconds and extension at 72ºC for 40 seconds. The final extension was carried out at 72ºC for seven minutes. The amplified products were separated on 2% agarose gel mixed with 5 ul/100 ml of ethidium bromide. The DNA bands were visualized and photographed using the gel documentation system (G:Box Syngene, USA). To amplify the target sequence of intron 13, thermal cycling conditions were as follows: the initial denaturation was at 95 ºC for three minutes. It was followed by 45 cycles of denaturation at 95 ºC for 35 seconds, annealing at 53ºC for 35 seconds and extension at 72^o^C for one minute. The final extension was at 72ºC for seven minutes. The quality of amplified products was checked on 3% agarose gel for dimers and non-specific bands. Due to technical difficulties of CA repeat analysis by PCR, standard Sanger sequencing method was opted to sequence the target templates.

### Statistical analysis:

Statistical Package for Social Sciences (SPSS) version 22.0 was used to analyze the data. Frequencies were calculated for categorical data and mean and standard deviation (SD) were calculated for continuous normally distributed data.

## RESULTS

A total of 80 “mother and son” pairs were studied. The subjects belonged to Rawalpindi district of Punjab province in Pakistan. The age of patients (son) ranged from 3 to 18 years having a mean age of 9.2 ± 4 years. The “mother and son” pairs were screened for F8 gene recurrent variants. A recurrent F8 gene variant was found in 32 pairs (40%). All the five F8 gene variants screened were found recurrent. Most recurrent variant was c.6869G>A. Frequency of each variant among patients and their mothers is shown in Table-II. All F8 variants detected were found both in mother and the sons. No two or more of screened variants were found in a single “mother and son” pairs. Fig.2 shows agarose gel photographs for c.1786T>C and c.1694G>A variant analysis.

**Table-II:** Haemophilia-A recurrent F8 variant analysis result.

F8 Variants	Variant type	Location	Protein	Frequency (patient)	Frequency (mother)
c.6869G>A	Nonsense	Exon 25	p.Trp2290*	12 (15%)	12 (15%)
c.1694G>A	Missense	Exon 11	p.(Gly565Glu)	08 (10%)	08 (10%)
c.1786T>C	Missense	Exon 12	p.(Ser596Pro)	06 (7.5%)	06 (7.5%)
c.195C>G	Nonsense	Exon 2	p.tyr65*	03 (3.75%)	03 (3.75%)
c.6972C>T	Nonsense	Exon 26	p.tyr 2324*	03 (3.75%)	03 (3.75%)
Overall frequency of the above variants				32 (40%)	32 (40%)
None of the above variants detected				48 (60%)	48 (60%)

**Fig.1:**
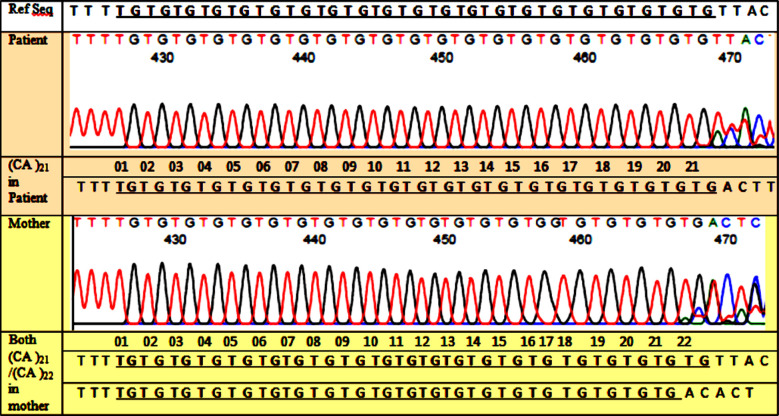
Sequencing result of intron 13 CA repeats in a Haemophilia-A family. The intron 13 CA^(n)^was informative because the mother has CA^(21)^ and CA^(22)^ repeats whereas the son (patient)has CA^(21)^.

**Fig.2:**
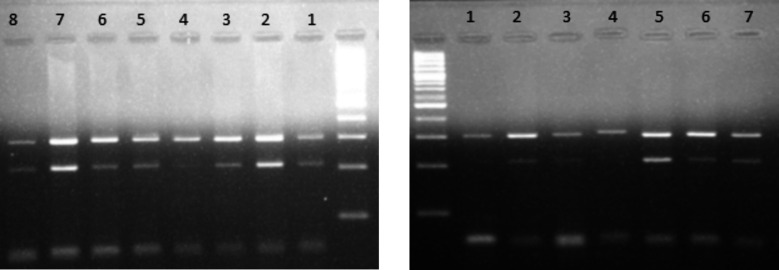
(A) Agarose gel photograph showing positive result forc.1786T>C in lane 2 and lane 7. The lane 1, 3, 4, 5, 6 and 8 shows negative result for c.1786T>C. (B). Agarose gel photograph showing positive result for c. 1694G>A in lane 5 and negative in lane 1, 2, 3, 4, 6 and 7.

Sanger sequencing of the CA repeats showed that these varied from CA^20^ to CA^23^. Fig.1 shows sequencing results of a family in which intron13 CA^n^ repeats were informative for linkage analysis Fig.2. Overall informativeness of intron 13 CA repeats of F8 gene among the “mother and son” pairs studied. Table-III

**Table-III:** Linkage based informativeness for intron 13 CA repeats of F8 gene through sequencing.

“mother and son” pairs	Polymorphic site	Type of Marker	Linkage established(informative)	Linkage not established(non-informative)
80	Intron 13 CA^(n)^	STR	29 “mother and son” pairs (36.2%)	51 “mother and son” pairs (63.8%)

## DISCUSSION

Haemophilia-A is one of the commonest inherited bleeding disorders. It is a single gene disorder resulting in deficiency or reduced plasma levels of factor VIII. Phenotypic severity is characterized by factor VIII plasma level: severe phenotype having plasma levels of <1%whereas moderate and mild phenotypes having plasma levels of 1-5% and 5-30% respectively.^10^

Keeping in view difficulties in management, high cost of treatment and low Health related quality of life of haemophilia patients, prevention of the disease is the most viable and practical option. Prevention through prenatal diagnosis by molecular diagnostic approaches includes linkage based analysis and direct variant analysis of disease causing variants.^11^ The gold standard for molecular diagnosis of Haemophilia-A is direct identification of the gene variant. Direct variant analysis of defective gene also bears certain limitations which includes; complexity and large size of factor VIII gene, variant heterogeneity, labor intensive and high cost. Keeping these limitations in mind this study is designed to look for frequency of reported F8 gene variants in Pakistani population having a causative or probable role in disease pathogenesis. A total of five F8 gene variants reported to be recurrent were screened in this study. The overall frequency of these variants was 40% in patients and their mothers. In a study conducted by Khanum et al.^10^ the overall frequency of these variants in Pakistani population was found to be 16.3%. The most likely reason for high prevalence of recurrent F8 gene variants in our study in comparison to the study conducted by Khanum et al.^10^ is the ethnicity of studied population. In study by Khanum et al.^10^ cohorts were recruited from all five provinces of Pakistan whereas in our study all subjects belong to Rawalpindi district of Punjab province of Pakistan.

The c.1786T>C and c.1694G>A are missense variants having a causative role in Haemophilia-A. In a study conducted by Khanum et al.^10^ these missense variants were screened in normal male Pakistani population to exclude possibility of these being neutral single nucleotide polymorphism (SNP) but they were found absent in studied population suggesting their pathogenic role. The c.6869G>A,c.195C>G and c.6972C>T are nonsense variant carrying high probability of pathogenic role.^9^c.1694G>A and c.195C>G are reported in patients having severe phenotype of Haemophilia-A whereas c.6869G>A is associated with moderate phenotype.^10^

In this study we have also analyzed CA repeats in intron 13 of F8 gene to determine its informativeness. Polymorphic markers are basically small DNA genetic sequence variant found in non-coding region of a gene. The informativity of each polymorphic marker varies among different ethnicity and populations. Therefore, it is essential to determine informativeness of these polymorphic markers related to factor VIII gene in a particular population.

Intron 13 CA repeats of F8 genewas foundinformative in 29 (36.2%) mother and son pairs in this study. The efficacy of this polymorphic marker varies in different ethnic groups. In studies conducted in India by Chowdhury et al^12^ and Jayandharan et al^13^ it was found to be informative in 51.2% and 56.6% of the studied population respectively. In Brazilian population and Japanese population intron 13 CA repeats is reported to be informative in 79% and 40% respectively.^14^,^15^ In another study conducted in Pakistan by Bugvi et al.^16^ on indirect linkage analysis, multiple highly informative polymorphic markers were used including intron 13 CA repeats. In this study intron 13 CA repeats were reported to be informative in 13 out of 22 families (59.1%). The informativeness of intron 13 CA repeats in our study is low in comparison to studies carried in India, Brazil and Japan. The most likely reason for this disparity is ethnicity of the studied population. One of the possible reasons for disparity of our results from Bugvi et al.^16^ is sample size. In our study 80 “mother and son” pairs were studied in comparison to 22 families studied by Bugvi et al.^16^ for same polymorphic marker.

In a recent study from Pakistan the reported minor allele frequency for intron 18/*Bc1*I and Intron 19/*Hind*III was 0.68 and 0.55 respectively.^17^ This study and our study provide the evidence that linkage analysis can serve as an effective and inexpensive diagnostic modality for prenatal diagnosis and carrier screening in Pakistani Haemophilia-A families. The informativeness of polymorphic markers will increase significantly on addition of intron 13 to intron 18 and intron 19 panel, since all these markers are informative in Pakistani population.

### Limitations:

Linkage based analysis though cost effective carries certain limitations that include: crossing over and recombination events among markers and variants can result in misdiagnosis and it constitutes 1% for intragenic and 5% for extragenic polymorphisms, non-availability of affected member or key members in family, somatic and germline mosaicism and non-informative polymorphic markers.

## CONCLUSION

The five known Haemophilia-A disease causing variants were found recurrent in Pakistani Haemophilia-A patients. Similarly, CA repeats in intron 13 of F8 gene was found to be informative in a significant proportion of the patients. These F8 gene variants and CA repeats in intron 13 of F8 gene can form a comprehensive basis for carrying out prenatal diagnosis in a significant number of Pakistani Haemophilia-A patients. At the same time other informative markers reported in Pakistani population on addition to this panel will make prenatal diagnosis a very effective, viable and cost-effective modality in Haemophilia-A disease prevention.

### Authors’ contribution:

**MAS** did data collection, statistical analysis, data interpretation, manuscript writing and is responsible for integrity of the research.

**SA** Conceived, Supervised the study project and approval of manuscript writing.

**MA and ST** did revision and editing of the manuscript.

## References

[1] Khalid K, Bilwani F, Adil SN, Khurshid M (2008). Frequency and clinical spectrum of rare inherited coagulopathies - A tricenter study. J Pak Med Assoc.

[2] Ahmed S, Ali W, Anwar M, Jameel T, Ayub M, Zafar T (1999). Prenatal Diagnosis of Haemophilia-A:A basis for the Pakistani Families. J Pak Med Assoc.

[3] Borhany M, Shamsi T, Moiz B, Hasan K, Hashmi KZ, Ayyub M (2012). Guidelines on the laboratory diagnosis of congenital bleeding disorders in Pakistan. J Pak Med Assoc.

[4] Bertamino M, Riccardi F, Banov L, Svahn J, Molinari AC (2017). Hemophilia Care in the Pediatric Age. J Clin Med.

[5] Goz M, Hazar A, Mordeniz C, Kocarslan A, Demirkol AH, Koc A (2009). Acute cardiac tamponade due to spontaneous bleeding in a child with Haemophilia A. J Pak Med Assoc.

[6] Peyvandi F, Jayandharan G, Chandy M, Srivastava A, Nakaya SM, Johnson MJ (2006). Genetic diagnosis of Haemophilia-And other inherited bleeding disorders. Haemophilia.

[7] Bowen DJ (2002). Haemophilia-A and haemophilia B:molecular insights. Mol Pathol.

[8] Chowdhury RM, Herrmann FH, Schroder W, Lalloz MRA, Layton M, Lambert C (2000). Factor VIII gene polymorphisms in Asian Indian population. Haemophilia.

[9] Afzal M, Rahim A, Naveed AK, Ahmed S, Kiyani MM (2018). Development of Cost-effective Tetra-primer Amplification Refractory Variant System (T-ARMS) PCR for the Detection of miR-146a gene rs2910164 C/G Polymorphism in Breast Cancer. Biochem Mol Biol J.

[10] Khanum F, Collins PW, Harris RL, Bowen DJ (2014). Characterization of F8 defects in Haemophilia-A in Pakistan:Investigation of correlation between variant type and the in vitro thrombin generation assay. Haemophilia.

[11] Khan MTM, Naz A, Ahmed J, Shamsi TS, Taj AS (2017). Diagnosis and phenotypic assessment of Pakistani Haemophilia B carriers. Pak J Med Sci.

[12] Chowdhury MR, Tiwari M, Kabra M, Menon PSN (2003). Prenatal diagnosis in hemophilia A using factor VIII gene polymorphism—Indian experience. Ann Hematol.

[13] Jayandharan G, Shaji RV, George B, Chandy M, Srivastava A (2004). Informativeness of linkage analysis for genetic diagnosis of Haemophilia-A in India. Haemophilia.

[14] de Carvalho FM, de Vargas Wolfgramm E, Paneto GG, de Paula Careta F, Spagnol Perrone AM, de Paula F (2007). Analysis of factor VIII polymorphic markers as a means for carrier detection in Brazilian families with Haemophilia-A. Haemophilia.

[15] Sawada A, Sumita C, Higasa S, Ueda M, Suehiro A, Kakishita E (2002). Suitability of four polymorphic DNA markers for indirect genetic diagnosis of Haemophilia-A in Japanese subject. Thromb Res.

[16] Bugvi SM, Imran M, Mahmood S, Hafeez R, Fatima W, Sohail S (2012). Screening of intron 1 inversion and three intragenic factor VIII gene polymorphisms in Pakistani hemophilia A families. Blood Coagul Fibrinolysis.

[17] Rasheed M, Asif N, Zawar A, Hassan K, Anwar T (2022). Indirect Genetic Analysis of Pakistani Hemophilia a Pedigrees by using BclI and HindIII Polymorphic Markers. J Haematol Stem Cell Res.

